# Feasibility and Impact of Out-of-Theatre Regional Anesthesia for Rib Fractures: A Retrospective Cohort Study

**DOI:** 10.7759/cureus.115690

**Published:** 2026-09-02

**Authors:** Salman Naeem, Luke Peacock, Syme Bhopal, Mehdin Shah, Ammar Habbal, James Bearman, Ahmed Hassan, Drew Gordon, Anna Vondy, Mohammad Dafalla, Grace Catchpole, Simrik Sunawar, Adriana Gonzalez, Hafsa Ali, Mahika Kamat, Muhammad K Buhadur Ali

**Affiliations:** 1 College of Medicine and Public Health, Flinders University, Adelaide, AUS; 2 Emergency Department, Flinders Medical Centre, Adelaide, AUS; 3 Department of Emergency Medicine, East Kent Hospitals University NHS Foundation Trust, Kent, GBR; 4 Department of Family Medicine, Medway Maritime Hospital, Kent, GBR; 5 Department of Anaesthesiology, East Kent Hospitals University NHS Foundation Trust, Kent, GBR; 6 Department of Acute Medicine, East Kent Hospitals University NHS Foundation Trust, Kent, GBR; 7 Department of Respiratory Medicine, The Grange University Hospital, Cwmbran, GBR; 8 Department of Anaesthesiology, Medway Maritime Hospital, Kent, GBR; 9 Department of Emergency Medicine, Warrington and Halton Teaching Hospitals NHS Foundation Trust, Warrington, GBR; 10 Department of Medicine, St. Richard's Hospital, Chichester, GBR; 11 Department of Acute Medicine, University Hospitals Plymouth NHS Trust, Plymouth, GBR; 12 Department of Medicine, King Edward Medical University, Lahore, PAK

**Keywords:** emergency department, out-of-theatre, regional anaesthesia, rib fracture, trauma

## Abstract

Introduction

Rib fractures cause significant morbidity due to pain-induced respiratory issues. While regional anesthesia (RA) provides excellent pain relief, evidence for its administration outside of theatres remains limited. This study aimed to evaluate the feasibility and clinical impact of out-of-theatre RA for traumatic rib fractures.

Methods

A retrospective cohort study was conducted at a district general hospital and regional trauma unit between January 2021 and December 2023. The primary outcomes were the feasibility and complications of RA. Secondary outcomes were mortality and length of stay (LOS).

Results

A total of 848 records were screened, and 682 patients met the inclusion criteria. Within this cohort, 126 received RA combined with systemic analgesia, while 556 received systemic analgesia alone. Out-of-theatre administration of RA was performed in 18.4% of patients, with the most common technique being the erector spinae plane block (ESPB). The majority of the blocks (83%) were performed by anesthetists. Complications included 26 (21%) instances of catheter dislodgement, 8 (16%) block failures, and one (1%) catheter infection. Overall mortality was 10%, with 10.4% in the systemic analgesia-only group and 7.9% in the RA with systemic analgesia group. The unadjusted OR for mortality with RA was 0.74 (95% CI, 0.36-1.49; p=0.4), and the adjusted OR was 0.85 (95% CI, 0.42-1.71; p=0.66), with neither reaching statistical significance. Median LOS was significantly longer in the RA group than in the systemic analgesia-only group: 9 days (IQR, 6-14) vs 6 days (IQR, 3-12), respectively (p<0.001), and remained so after adjustment.

Conclusion

Out-of-theatre RA for rib fractures may be feasible, with relatively few reported complications in our cohort. RA was delivered using fascial plane blocks. However, it was not possible to associate the use of RA with mortality or LOS due to the limitations of this study.

## Introduction

Acute rib fractures are rib fractures resulting from acute trauma, although there is currently no consensus definition. They can result from blunt and penetrating chest trauma and are associated with significant morbidity and mortality [1]. The incidence of rib fractures is estimated to be 30 per 100,000 person-years in females and 46 per 100,000 person-years in males, with an increasing incidence in females due to falls [1]. In the United Kingdom (UK), the most common mechanisms of injury leading to rib fractures are low-energy transfer falls from less than two metres and high-energy transfer trauma, mainly from road traffic collisions [2].

The increasing burden of rib fractures falls disproportionately on older patients. Frailty, which is measured using tools such as the Clinical Frailty Scale or Rockwood Frailty Score (RFS), independently predicts poor outcomes following thoracic trauma, compounding the risks associated with age and fracture burden [3]. This population is particularly vulnerable to the adverse effects of systemic analgesics and is least able to tolerate the consequences of inadequate pain control, making the choice of analgesic strategy especially important in this group [4].

Mortality from rib fractures is approximately 10% across all ages [5]. The most significant factors associated with mortality are age greater than 65 years and the number of ribs fractured; patients aged 65 years or older have up to five times higher odds of dying compared with those under the age of 65 years [5,6]. Significant morbidity exists 6-12 months after rib fractures, including new chronic pain (43%), increased limitation of daily activities (29%) in people aged over 65 years, physical limitations (56%), and inability to return to work (28%) [7]. Morbidity and mortality are largely due to pain-induced hypoventilation, pneumonia, and respiratory failure [4]. The mainstay of treatment is ensuring effective analgesia, which is essential to reduce the sequence of events leading to complications and death [4,5]. Identifying patients at greatest risk of morbidity and mortality is crucial; numerous risk stratification tools exist for predicting outcomes from rib fractures, including the STUdy of the Management of BLunt chest wall trauma (STUMBL) score and Pain, Inspiratory Capacity, and Cough (PIC) score [8,9]. These tools can allow prognostication and stratification of patients at highest risk of mortality and morbidity and facilitate targeted management.

Effective analgesia is essential in the management of rib fractures. Immobilisation is generally employed as a non-pharmacological approach to analgesia in fracture management [10]. Rib fractures are unique in that they must move 12-20 times per minute to ensure ventilation and cannot be immobilised without surgical intervention. Surgical stabilisation of rib fractures (SSRF) is an option in selected patients with flail chest or severely displaced fractures and has been shown to reduce ventilator dependence and improve respiratory mechanics [11]. However, the majority of patients, especially the elderly and frail, are not surgical candidates, and non-operative analgesic management remains the cornerstone of care for most [12]. Multimodal analgesia is crucial to prevent hypoventilation and subsequent sequelae leading to morbidity and mortality [4]. Systemic analgesia typically involves regular acetaminophen, non-steroidal anti-inflammatory drugs (NSAIDs), and oral opioids, with escalation to IV opioids or patient-controlled analgesia (PCA) if required. In older populations, systemic analgesics, particularly opioids and NSAIDs, increase the risk of constipation, respiratory depression, over-sedation, hypotension, delirium, renal dysfunction, and gastrointestinal bleeding [13]. Regional anesthesia (RA) techniques reduce opioid use and provide superior analgesia compared with systemic analgesia alone; depending on the site of fracture, these include epidural analgesia (EA), paravertebral block (PVB), erector spinae plane block (ESPB), serratus anterior plane block (SAPB), and intercostal block (ICB) [14,15]. RA has been found to reduce the odds of mortality when compared with systemic analgesia in elderly patients [16,17]. Whilst thoracic epidural anesthesia has traditionally been the gold standard for analgesia in rib fractures, fascial plane techniques are rapidly becoming more widely adopted [18,19]. RA techniques reduce pain scores and improve respiratory function in the context of multiple rib fractures, and certain fascial plane techniques have been shown to be safe and effective modes of analgesia, even in selected patients with coagulopathy or systemic infection [20-23].

There remains a significant evidence gap regarding the feasibility and safety of RA delivered outside of the operating theatre, i.e., performed at the bedside, in the ED, or on the ward. Real-world data on out-of-theatre delivery, including who performs it, under what governance structures, and with what complication profile, remain insufficient. Furthermore, whether RA delivered in this less controlled setting achieves comparable clinical outcomes to theatre-based RA, and how it compares with systemic analgesia alone in unselected trauma populations, has not been adequately characterised. Patient-tailored multimodal analgesia is a recommended approach for the management of rib fractures; however, there remains a need for further studies of regional anaesthetic techniques for the management of acute rib fractures [18]. This study aims to provide evidence for the feasibility and impact of out-of-theatre RA for traumatic rib fractures.

## Materials and methods

A retrospective cohort study was conducted at a district general hospital and regional trauma unit between January 2021 and December 2023. The study did not meet the Health Research Authority (HRA) criteria for a formal research study and was registered with the local audit department as a service evaluation [24]. The study adheres to the Strengthening the Reporting of Observational Studies in Epidemiology (STROBE) guidelines for reporting observational studies [25].

Setting

The study was conducted at a regional trauma unit in the United Kingdom, serving a population of approximately 654,000. The hospital provided intensive care and general and specialised surgical services. However, it did not have cardiothoracic surgical services. A pathway for the management of thoracic injuries was established in 2020 and is provided in Appendix 1. Patients received systemic analgesia as part of the normal standard of care. However, patients scoring high on the modified PIC score were assessed for RA, which was provided by both anesthetists and emergency physicians at the bedside. The patients had RA catheters inserted either in the ED or on the ward. Patients were transferred to the major trauma centre if they met the major trauma criteria, required specialised cardiothoracic input, or required surgical fixation of ribs as defined in the pathway (Appendix 1).

Data acquisition and management

All data were retrieved from the Electronic Medical Records (EMR) system (Sunrise). A list of all patients with a primary or secondary diagnosis of rib fractures was compiled. All patients who were admitted to the hospital with at least one acute rib fracture, defined as a rib fracture within one week of injury or defined by the radiologist as an acute rib fracture on CT scan of the chest or chest X-ray, were included. Patients who were transferred to or from the hospital, had old rib fractures (>7 days), or had missing core data, i.e., demographics, site and time of injury, and analgesia received, were excluded. A dedicated team extracted and analysed data from the patients' EMR.

For each patient, a standardised dataset was collected, including demographic information, co-morbidities, resuscitation status, mechanism of injury, day and time of presentation, number of ribs fractured, imaging modality used for diagnosis, frailty score, mortality, length of hospital stay (LOS), grade of the reviewing clinician, injuries sustained, analgesics used, use of RA, the specialty of the individual performing the RA, and any associated complications. All collected data were anonymised and encrypted for secure storage and analysis. Data were extracted and analysed using SPSS 29.0 (IBM SPSS Statistics, IBM Corporation, Chicago, IL, USA).

Outcomes

The primary outcomes of the study were the feasibility and complications of RA performed out of theatre, while the secondary outcome was to assess its clinical impact by assessing the effect of RA use on mortality and LOS. Feasibility was assessed by the percentage of patients receiving RA and the rate of complications. Complications included catheter dislodgement, defined as the catheter falling out of the site of insertion; block failure, defined as failure of the block to provide anaesthesia/analgesia to the area it would cover; and infection, defined as signs of hyperaemia, pain, purulent discharge, and/or the clinician's suspicion of infection. Statistical analysis utilised chi-square tests and ORs to assess crude associations with mortality and the Mann-Whitney U test to evaluate differences in LOS between groups. Multivariate binary logistic regression was performed to identify independent predictors of mortality, with adjusted ORs reported. A generalised linear model with a gamma distribution and log link was used to evaluate adjusted LOS.

Statistical analysis

All statistical analyses were performed using SPSS 29.0 (IBM SPSS Statistics, IBM Corporation, Chicago, IL, USA). Normality was assessed using the Shapiro-Wilk test. Non-normally distributed continuous data were reported using median (IQR) and categorical data as frequency and percentage, while normally distributed continuous data were reported using mean (SD). Participant characteristics were compared using the chi-square and Kruskal-Wallis tests for significance, as appropriate. The strength of association between RA and mortality was quantified by the unadjusted OR and adjusted odds ratio (aOR), with 95% CI, using multivariate binary logistic regression to account for covariates. Key variables that were significantly different between the two groups were considered (i.e., RFS [26] on admission, age, trauma call activation, number of ribs fractured, lung contusion, pneumothorax, flail segment, haemothorax, subcutaneous emphysema, use of NSAIDs, opioids, and PCA). The model identified age, number of ribs fractured, RFS on admission, and use of PCA as independent risk factors associated with mortality. All variables except the use of PCA were included in the final multivariable regression analysis due to ambiguity regarding the duration of PCA use. The Mann-Whitney U test was performed to calculate unadjusted LOS. RFS on admission, age, and number of ribs fractured were used in generalised linear modelling with a gamma distribution and log link to adjust for covariates. A p-value less than 0.05 was considered statistically significant.

## Results

We extracted 848 records with a primary or secondary diagnosis of rib fractures from the hospital database. The records were screened, and 682 were included for review after meeting the predefined inclusion and exclusion criteria, as shown in Figure 1.

**Figure 1:**
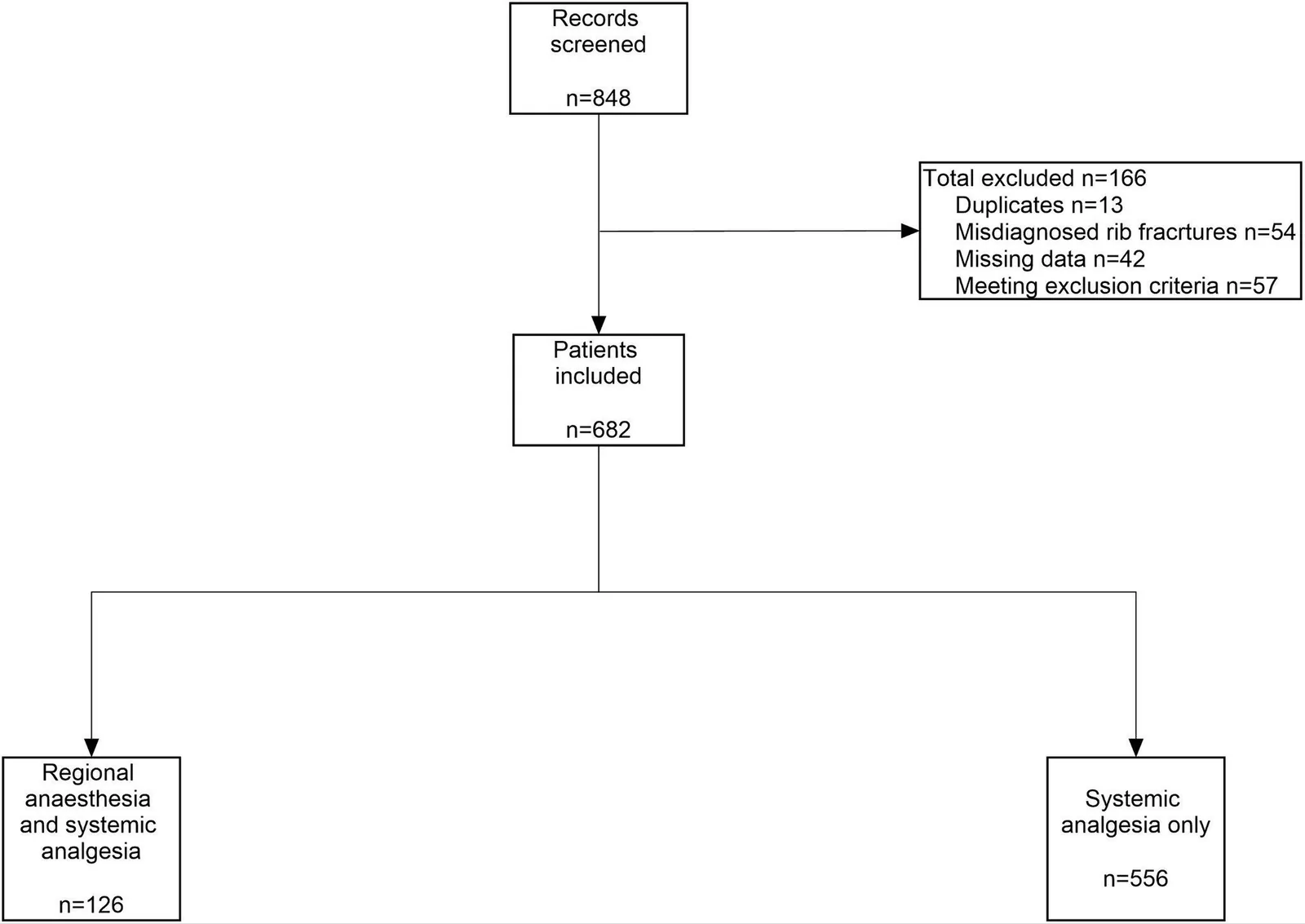
Flow diagram of patient selection, exclusions, and allocation to treatment groups.

Basic demographic characteristics, along with the significance of differences between the two groups, are described in Table 1.

**Table 1:** Baseline characteristics, injury mechanisms, and clinical outcomes of patients with rib fractures receiving regional anaesthesia with systemic analgesia or systemic analgesia alone. ACP: Advanced clinical practitioner; COPD: Chronic obstructive pulmonary disease; DNACPR: Do not attempt cardiopulmonary resuscitation; RA: Regional anaesthesia; RFS: Rockwood Frailty Score; RTC: Road traffic collision. Note: Values are presented as n (%), mean (SD), or median (IQR), as appropriate. p-values represent between-group comparisons.

Characteristic	Total (N=682)	RA and systemic analgesia (n=126)	Systemic analgesia only (n=556)	p value	Statistical test, statistic, and effect size
Demographics					
Age, years, mean (SD)	69 (17.3)	69 (16.0)	69 (17.6)	0.99	Independent-samples Student’s t-test; t(680)=0.013; Cohen’s d=0.001
Male sex, n (%)	413 (61)	77 (61)	336 (60)	0.888	Pearson’s χ² test; χ²(1)=0.020; Cramér’s V=0.005
DNACPR, n (%)	112 (16)	17 (14)	95 (17)	0.325	Pearson’s χ² test; χ²(1)=0.967; Cramér’s V=0.038
Comorbidities, n (%)					
Hypertension	281 (41)	57 (45)	224 (40)	0.308	Pearson’s χ² test; χ²(1)=1.039; Cramér’s V=0.039
Diabetes	106 (16)	16 (13)	90 (16)	0.329	Pearson’s χ² test; χ²(1)=0.952; Cramér’s V=0.037
Osteoporosis	85 (13)	18 (14)	67 (12)	0.493	Pearson’s χ² test; χ²(1)=0.471; Cramér’s V=0.026
Cancer	89 (13)	13 (10)	76 (14)	0.313	Pearson’s χ² test; χ²(1)=1.017; Cramér’s V=0.039
COPD	93 (14)	22 (17)	71 (13)	0.166	Pearson’s χ² test; χ²(1)=1.919; Cramér’s V=0.053
Obstructive sleep apnoea	12 (2)	2 (2)	10 (2)	1	Fisher’s exact test; OR=0.88 (95% CI, 0.09-4.21)
Falls within last 6 months, n (%)	195 (29)	23 (18)	172 (31)	0.004	Pearson’s χ² test; χ²(1)=8.091; Cramér’s V=0.109
Mechanism of injury, n (%)					
Fall from standing	280 (41)	53 (42)	227 (40)	0.32	Pearson’s χ² test (overall); χ²(7)=8.141; Cramér’s V=0.109
Fall from height >1 metre	161 (24)	32 (25)	129 (23)		
Fall from sitting/lying	20 (3)	4 (3)	16 (3)		
RTC <30 mph	53 (8)	13 (10)	40 (7)		
RTC >30 mph	77 (11)	15 (12)	62 (11)		
Cardiac arrest	18 (3)	0 (0)	18 (3)		
Assault	17 (3)	1 (1)	16 (3)		
Other	56 (8)	8 (6)	48 (9)		
Rockwood Frailty Score, median (IQR)					
On admission	3 (2-5)	3 (1-4)	3 (2-5)	0.03	Mann-Whitney U test
At discharge	4 (2-6)	4 (2-6)	4 (2-6)	0.23	Mann-Whitney U test
Presentation characteristics, n (%)					
Weekday admission	482 (71)	86 (68)	396 (71)	0.509	Pearson’s χ² test; χ²(1)=0.437; Cramér’s V=0.025
Weekend admission	200 (29)	40 (32)	160 (29)		
Admission 0800-2000	451 (66)	86 (68)	365 (66)	0.577	Pearson’s χ² test; χ²(1)=0.312; Cramér’s V=0.021
Admission 2000-0800	231 (34)	40 (32)	191 (34)		
Trauma call	259 (38)	54 (43)	205 (37)	0.211	Pearson’s χ² test; χ²(1)=1.563; Cramér’s V=0.048
Grade of initial clinician, n (%)					
ACP	15 (2)	3 (2)	12 (2)	0.984	Pearson’s χ² test (overall); χ²(3)=0.161; Cramér’s V=0.015
Junior doctor	177 (26)	31 (25)	146 (26)		
Registrar/ST4 and above	405 (59)	76 (60)	329 (59)		
Consultant	85 (12)	16 (13)	69 (12)		
Outcomes					
Lower respiratory tract infection, n (%)	107 (16)	19 (15)	88 (16)	0.835	Pearson’s χ² test; χ²(1)=0.043; Cramér’s V=0.008
Hyponatraemia, n (%)	26 (4)	3 (2)	23 (4)	0.448	Fisher’s exact test; OR=0.57 (95% CI, 0.11-1.92)
Delirium, n (%)	56 (8)	17 (14)	39 (7)	0.017	Pearson’s χ² test; χ²(1)=5.719; Cramér’s V=0.092
Constipation, n (%)	67 (10)	17 (14)	50 (9)	0.126	Pearson’s χ² test; χ²(1)=2.347; Cramér’s V=0.059
Length of stay, days, median (IQR)	6 (4-13)	9 (6-14)	6 (3-12)	<0.001	Mann-Whitney U test; U=26,200; rank-biserial r=0.252
Mortality, n (%)	68 (10)	10 (7.9)	58 (10.4)	0.399	Pearson’s χ² test; χ²(1)=0.712; Cramér’s V=0.032

Injury characteristics and analgesics used

The median number of rib fractures was 3 (IQR, 2-4), and 598 (87%) patients had unilateral rib fractures. A significant proportion, 399 (58%), of patients had isolated thoracic injuries. Patients who received RA and systemic analgesia had a significantly higher number of rib fractures and other thoracic injuries, including lung contusion, pneumothorax, subcutaneous emphysema, flail segment, and haemothorax. Paracetamol and opioids were the most frequently used analgesics (93% (n=634) and 87% (n=593), respectively); however, systemic analgesic use differed significantly between the two groups. The injury characteristics and analgesics used are summarised in Table 2.

**Table 2:** Injury characteristics, associated thoracic and non-thoracic injuries, and systemic analgesic use in patients receiving regional anaesthesia with systemic analgesia compared with those receiving systemic analgesia alone. NSAIDs: Non-steroidal anti-inflammatory drugs; PCA: Patient-controlled analgesia; RA: Regional anaesthesia. Note: Values are presented as n (%) or median (IQR), as appropriate. p-values represent between-group comparisons.

Characteristic	Total (N=682)	RA and systemic analgesia (n=126)	Systemic analgesia only (n=556)	p-value	Statistical test, statistic, and effect size
Rib fractures					
Number, median (IQR)	3 (2-4)*	4 (3-6)	3 (3-4)	<0.001	Mann-Whitney U test
Laterality, n (%)					
Unilateral	595 (87)	114 (90)	481 (87)	0.228	Pearson’s χ² test (overall); χ²(1)=1.451; Cramér’s V=0.046
Bilateral	87 (13)	12 (10)	75 (13)		
Other thoracic injuries, n (%)					
Lung contusion	103 (15)	33 (26)	70 (13)	<0.001	Pearson’s χ² test; χ²(1)=14.819; Cramér’s V=0.147
Pneumothorax	153 (22)	54 (43)	99 (18)	<0.001	Pearson’s χ² test; χ²(1)=37.046; Cramér’s V=0.233
Flail segment	30 (4)	14 (11)	16 (3)	<0.001	Pearson’s χ² test; χ²(1)=16.559; Cramér’s V=0.156
Haemothorax	88 (13)	27 (21)	61 (11)	0.002	Pearson’s χ² test; χ²(1)=9.996; Cramér’s V=0.121
Subcutaneous emphysema	75 (11)	31 (25)	44 (8)	<0.001	Pearson’s χ² test; χ²(1)=29.233; Cramér’s V=0.207
Injuries other than thoracic injuries, n (%)	283 (42)	55 (44)	228 (41)	0.587	Pearson’s χ² test; χ²(1)=0.296; Cramér’s V=0.021
Analgesia, n (%)					
Paracetamol	634 (93)	121 (96)	513 (92)	0.136	Pearson’s χ² test; χ²(1)=2.226; Cramér’s V=0.057
NSAIDs	202 (30)	47 (37)	155 (28)	0.036	Pearson’s χ² test; χ²(1)=4.376; Cramér’s V=0.080
Opioids	593 (87)	121 (96)	472 (85)	<0.001	Pearson’s χ² test; χ²(1)=11.234; Cramér’s V=0.128
PCA	358 (52)	104 (83)	254 (46)	<0.001	Pearson’s χ² test; χ²(1)=55.953; Cramér’s V=0.286

Regional anaesthesia

RA was used in combination with systemic analgesia in 126 patients (18%). The majority of patients received fascial plane blocks, i.e., ESP, SAPB, and PVB. The majority of blocks (n=104, 83%) were performed by anaesthetists. The types of blocks, specialties performing the blocks, and complications of the blocks are summarised in Table 3.

**Table 3:** Details of regional anaesthesia administration, including block types, performing specialties, and associated complications. RA: Regional anaesthesia.

Characteristic	RA and systemic analgesia (n=126)
Type of regional anaesthesia administered, n (%)	
Epidural	19 (15)
Erector spinae plane block	83 (66)
Paravertebral block	1 (1)
Serratus anterior plane block	20 (16)
Other	3 (2)
Specialty performing regional anaesthesia, n (%)	
Anaesthesia	104 (83)
Emergency medicine	22 (17)
Complications, n (%)	
Catheter dislodgement	26 (21)
Failure of block	8 (6)
Infection	1 (1)

Radiological investigations

The initial radiological investigation was CT of the chest in 552 (80%) patients, whilst 661 (97%) of the included population underwent CT of the chest to confirm the diagnosis of rib fractures. CT examinations were reported by in-house radiologists in 375 (55%) patients. The location of rib fractures along the rib was reported in 547 (80%) patients, whilst the site and number of fractured ribs were reported in 635 (93%) patients.

Clinical outcomes

Overall mortality in this cohort was 10%, with 10.4% in the systemic analgesia-only group and 7.9% in the combined RA and systemic analgesia group. The unadjusted OR for mortality with RA and systemic analgesia was 0.74 (95% CI, 0.36-1.49; p=0.40) and did not reach statistical significance. After adjusting for age, number of ribs fractured, and RFS on admission using multivariable binary logistic regression, the aOR for mortality with RA and systemic analgesia was 0.85 (95% CI, 0.42-1.71; p=0.66).

LOS was significantly longer in patients who received RA compared with those who did not (median (IQR): 9 (6-14) vs 6 (3-12) days; Mann-Whitney U=26,200; p<0.001). A generalised linear model using a gamma distribution with a log link was used to assess predictors of LOS. After adjusting for age, frailty, and number of ribs fractured, RA was associated with a significantly longer hospital stay (adjusted LOS ratio, 1.16; 95% CI, 1.05-1.28; p=0.004). Age, frailty, and number of fractured ribs were independent predictors of prolonged LOS (p<0.01 for all). The model demonstrated good overall fit (deviance/df=1.03).

## Discussion

In this retrospective cohort study, we evaluated the feasibility and clinical impact of out-of-theatre RA in patients with traumatic rib fractures and highlighted its real-world application within a modern trauma care pathway. Nearly all patients underwent chest CT imaging, reflecting reliance on advanced imaging for accurate diagnosis and injury characterisation. For subsequent pain management, fascial plane blocks, particularly ESPB, SAPB, and PVB, were the predominant techniques, most commonly performed by anaesthetists and EM physicians, illustrating a shift toward safer, ultrasound-guided RA delivered outside theatres and integrated into trauma care. Notably, thoracic EA, historically the gold standard, was used in only 19 patients (15%) in this cohort, a proportion that reflects the practical limitations of epidural delivery in an acute trauma population. Coagulopathy from haemorrhage or anticoagulant use, haemodynamic instability, and the technical demands of epidural insertion outside a controlled theatre environment collectively limit its applicability, and this distribution of block types reflects a pragmatic, patient-tailored approach rather than a departure from best practice [27].

Traditionally, RA has been delivered in operating theatres, where controlled environments, dedicated staffing, and established aseptic protocols are readily available. However, with an ageing population and rising volumes of elderly trauma patients, theatre capacity has become an increasingly scarce resource. This pressure has accelerated the shift toward delivering RA outside the operating theatre to ensure timely and effective analgesia for patients with rib fractures and other painful injuries. Importantly, published evidence does not demonstrate a corresponding rise in complication rates, supporting the safety of out-of-theatre RA when appropriate systems are in place [23]. In this cohort, out-of-theatre delivery was embedded within a structured institutional pathway incorporating modified PIC score-based triage, shared governance between anaesthetics and emergency medicine (EM), and defined protocols for catheter management and complication response, components that published evidence identifies as prerequisites for safe out-of-theatre RA [28].

As the delivery of out-of-theatre RA expands, EM physicians have taken on a progressively larger role, demonstrating that they can deliver RA safely, with overall complication rates reported to be as low as 0.4% [29,30]. Our findings reflect this strong safety profile, as we observed only a single case of infection (1%), aligning with published literature in which infection rates remain well under 1% for single-shot blocks and 0-3% for continuous catheters managed with appropriate asepsis [28]. Maintaining these low complication rates and ensuring patient safety rely fundamentally on establishing shared governance between EM and anaesthetics departments to ensure that all clinicians are appropriately trained. Furthermore, successful implementation requires strict adherence to clear protocols for catheter management and meeting crucial logistical requirements, including access to portable ultrasound, sterile equipment, and clean procedural areas. Finally, to mitigate out-of-theatre risks, resuscitation facilities with appropriate monitoring and lipid emulsion availability must be immediately accessible to manage potential complications such as local anaesthetic systemic toxicity (LAST).

Use of ESPB and SAPB for rib fracture analgesia has increased in recent years due to their relative simplicity, safety, and ultrasound-guided precision [23]. Both techniques provide effective pain relief, with several RCTs and observational studies reporting reduced opioid use and significant pain reduction, particularly with SAPB, though evidence from ESPB versus PVB trials remains mixed regarding broader clinical outcomes, including mortality benefits [15,31,32]. While some studies have shown potential advantages such as lower mortality and reduced delirium, larger meta-analyses and studies have failed to demonstrate consistent survival benefits [33,34]. A recent study using Trauma Quality Improvement Program (TQIP) data found no difference in mortality rates and longer hospital stays among RA recipients [35].

Although crude mortality appeared lower among patients who received RA (7.9% compared with 10.4% in the systemic analgesia-only group), this difference was not statistically significant after adjustment for age, number of rib fractures, and frailty, indicating a lack of association between the use of RA and mortality benefit. This finding is consistent with previous work [33-35]. In contrast, older age, greater fracture number, and higher RFS were confirmed as significant predictors of mortality, consistent with established evidence [36]. Several factors may explain the absence of a significant mortality effect, such as the relatively small and asymmetric sample sizes. While not statistically significant, the lower mortality rate observed in such a high-risk group is promising and warrants further investigation in larger studies.

Regarding LOS, patients who received RA stayed approximately three days longer than those managed without RA (median, 9 vs 6 days). This association remained significant even after risk adjustment (adjusted LOS ratio, 1.16). This finding carries implications that deserve acknowledgement; a longer hospital stay creates a clinically and economically significant strain on bed occupancy, especially for district general hospitals that are already operating under capacity pressure.

A difference in delirium rates was also observed, with the RA group demonstrating higher rates compared with the systemic analgesia-only group (14% vs 7%, p=0.017). This finding is seemingly at odds with existing literature, including work by O'Connell KM et al., who found that regional analgesia was associated with reduced delirium risk in geriatric trauma patients with multiple rib fractures [33]. However, in the context of this cohort, this apparent paradox may be attributable to confounding by indication along with other confounders. Patients who received RA had a significantly greater burden of thoracic injury, with higher rates of pneumothorax, lung contusion, and flail segments, all of which independently predispose to delirium through hypoxia, inflammation, and prolonged hospitalisation. As such, this finding should be interpreted with caution and does not negate the potential delirium-reducing mechanisms of RA.

Both findings, the prolonged LOS and the absence of an independent survival benefit, are possibly a result of confounding by indication. The observed differences in injury severity between groups, especially the greater number of rib fractures and higher prevalence of thoracic injuries in the RA group, suggest that RA was selectively administered to patients with more significant trauma. Systemic analgesic use was also higher among patients who received RA, with greater use of NSAIDs, opioids, and PCA. This likely reflects either the greater pain burden arising from more severe injuries in this group or a step-up approach in which patients were first trialled on systemic analgesics before being considered for RA. Therefore, it is possible that LOS may be determined by factors unrelated to RA itself, as we did not capture the timing of RA administration or the duration of its use, and patients with a higher injury burden may require additional interventions, such as surgery, intensive physiotherapy, or experience delays related to rehabilitation placement. While this does not argue against RA adoption, commissioners and clinical leads implementing out-of-theatre RA pathways should be aware that anticipated efficiency gains (i.e., shorter stays) may not be immediately realised in heterogeneous, unselected trauma populations, underscoring the need for prospective evaluation alongside clinical outcomes.

Overall, these findings underscore that while RA represents an important component of multimodal analgesia for rib fractures, its independent impact on mortality and broader recovery outcomes remains uncertain. Further prospective, adequately powered studies are warranted to clarify whether RA provides a true survival or recovery benefit within this heterogeneous and often frail patient population.

This study has several limitations that should be considered when interpreting its findings. Its retrospective cohort design limits the ability to draw causal inferences between RA and clinical outcomes. The relatively small sample size further reduces statistical power, particularly for detecting differences in outcomes such as mortality. The lack of randomisation also introduces clear selection bias and confounding by indication, given that patients receiving RA typically presented with a more severe injury burden that could skew clinical outcomes. Moreover, being a single-centre study, the findings may not be generalisable to other institutions with different patient populations or resources. There was also a lack of standardisation in the timing, type, and technique of RA, as well as variability in clinician expertise, which may affect both efficacy and complication rates. The study did not capture the duration of regional or systemic analgesia use, making it difficult to fully evaluate their independent effects on outcomes such as LOS. Due to the small sample size, all RA techniques were pooled together to assess their effect on mortality and LOS. This introduces bias, as each technique has different indications and may variably affect analgesia. The absence of control over concurrent treatments makes it difficult to evaluate the independent effect of RA. Finally, validated pain scores such as the Numerical Rating Scale (NRS) or Visual Analogue Scale (VAS) were not systematically captured in the electronic records, precluding assessment of analgesic efficacy as a direct outcome measure.

## Conclusions

This study shows that out-of-theatre RA may be a feasible and safe intervention for traumatic rib fractures. The shift towards ultrasound-guided fascial plane blocks, delivered primarily by anesthetists and emergency physicians, reflects a practical adaptation to constrained theatre capacity without compromising patient safety. The effects of bias and confounding due to the small sample size and other confounders could not be completely eliminated. Hence, even after adjusting for covariates such as age, frailty, and number of ribs fractured, RA was not independently associated with improved survival or LOS. To account for these limitations, future prospective trials are required to accurately evaluate the efficacy of out-of-theatre RA.

## Figures and Tables

**Figure 2:**
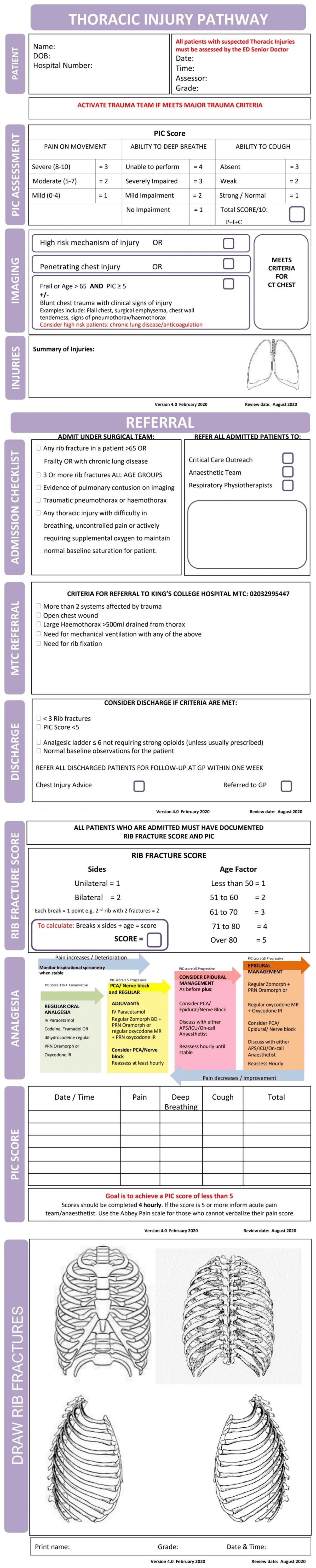
Thoracic injury pathway used in this study. Rib fracture score adapted from Easter A (2001) [37]. PIC score adapted from Bass GA et al. (2022) [38]. PIC: Pain, Inspiration and Cough; MTC: Major Trauma Centre; PCA: Patient-controlled analgesia; BD: bis in die (twice a day); PRN: pro re nata (as needed); MR: Modified release; IR: Immediate release; APS: Acute Pain Service.

## Appendices

Appendix 1

## References

[1] Proctor DW, Goodall R, Borsky K, Salciccioli JD, Marshall DC, Shalhoub J (2024). Trends in the incidence of rib and sternal fractures: a nationwide study of the global burden of disease database, 1990-2019. Injury.

[2] Ingoe HM, Eardley W, McDaid C, Rangan A, Lawrence T, Hewitt C (2020). Epidemiology of adult rib fracture and factors associated with surgical fixation: analysis of a chest wall injury dataset from England and Wales. Injury.

[3] Mohseni S, Forssten MP, Mohammad Ismail A, Cao Y, Hildebrand F, Sarani B, Ribeiro MA Jr (2024). Investigating the link between frailty and outcomes in geriatric patients with isolated rib fractures. Trauma Surg Acute Care Open.

[4] Coary R, Skerritt C, Carey A, Rudd S, Shipway D (2020). New horizons in rib fracture management in the older adult. Age Ageing.

[5] Piennette PD, Morris SE, Northern T (2022). Current review of analgesia and regional anesthesia practices for rib fracture and chest wall trauma. Curr Anesthesiol Rep.

[6] Bergeron E, Lavoie A, Clas D (2003). Elderly trauma patients with rib fractures are at greater risk of death and pneumonia. J Trauma.

[7] Heindel P, Ordoobadi A, El Moheb M (2022). Patient-reported outcomes 6 to 12 months after isolated rib fractures: a nontrivial injury pattern. J Trauma Acute Care Surg.

[8] Battle C, Hutchings H, Lovett S (2014). Predicting outcomes after blunt chest wall trauma: development and external validation of a new prognostic model. Crit Care.

[9] Terry SM, Shoff KA, Sharrah ML (2021). Improving blunt chest wall injury outcomes: introducing the PIC score. J Trauma Nurs.

[10] Mota M, Santos E, Cunha M, Abrantes T, Caldes P, Santos MR (2021). Non-pharmacological interventions for acute pain management in adult trauma victims: a scoping review. JBI Evid Synth.

[11] Sharma VJ, Summerhayes R, Wang Y, Kure C, Marasco SF (2024). Surgical stabilisation of rib fractures: a meta-analysis of randomised controlled trials. Injury.

[12] Shiroff AM, Wolf S, Wu A, Vanderkarr M, Salonga R, Galvain T, Holy CE (2022). Surgical fixation of severe rib fractures: a systematic literature review and meta-analysis. Arch Orthop.

[13] O'Neil CK, Hanlon JT, Marcum ZA (2012). Adverse effects of analgesics commonly used by older adults with osteoarthritis: focus on non-opioid and opioid analgesics. Am J Geriatr Pharmacother.

[14] Gamberini L, Moro F, Dallari C (2025). Regional anesthesia modalities in blunt thoracic trauma: a systematic review and Bayesian network meta-analysis. Am J Emerg Med.

[15] Partyka C, Asha S, Berry M (2024). Serratus anterior plane blocks for early rib fracture pain management: the SABRE randomized clinical trial. JAMA Surg.

[16] Malekpour M, Hashmi A, Dove J, Torres D, Wild J (2017). Analgesic choice in management of rib fractures: paravertebral block or epidural analgesia?. Anesth Analg.

[17] Womack J, Pearson JD, Walker IA, Stephens NM, Goodman BA (2019). Safety, complications and clinical outcome after ultrasound-guided paravertebral catheter insertion for rib fracture analgesia: a single-centre retrospective observational study. Anaesthesia.

[18] El-Boghdadly K, Wiles MD (2019). Regional anaesthesia for rib fractures: too many choices, too little evidence. Anaesthesia.

[19] Victorino GP, Chong TJ, Pal JD (2003). Trauma in the elderly patient. Arch Surg.

[20] Beard L, Hillermann C, Beard E, Millerchip S, Sachdeva R, Gao Smith F, Veenith T (2020). Multicenter longitudinal cross-sectional study comparing effectiveness of serratus anterior plane, paravertebral and thoracic epidural for the analgesia of multiple rib fractures. Reg Anesth Pain Med.

[21] White LD, Riley B, Davis K (2021). Safety of continuous erector spinae catheters in chest trauma: a retrospective cohort study. Anesth Analg.

[22] Adhikary SD, Liu WM, Fuller E, Cruz-Eng H, Chin KJ (2019). The effect of erector spinae plane block on respiratory and analgesic outcomes in multiple rib fractures: a retrospective cohort study. Anaesthesia.

[23] Jiang M, Peri V, Ou Yang B, Chang J, Hacking D (2023). Erector spinae plane block as an analgesic intervention in acute rib fractures: a scoping review. Local Reg Anesth.

[24] Gibbard E, Brandling J, Harding T. Best Practice in the Ethics and Governance of Service Evaluation (2017). Best Practice in the Ethics and Governance of Service Evaluation: Guidelines for evaluators and commissioners of evaluation in health and social care. Group.

[25] von Elm E, Altman DG, Egger M, Pocock SJ, Gøtzsche PC, Vandenbroucke JP (2008). The Strengthening the Reporting of Observational Studies in Epidemiology (STROBE) statement: guidelines for reporting observational studies. J Clin Epidemiol.

[26] Rockwood K, Song X, MacKnight C, Bergman H, Hogan DB, McDowell I, Mitnitski A (2005). A global clinical measure of fitness and frailty in elderly people. CMAJ.

[27] Wu JJ, Lollo L, Grabinsky A (2011). Regional anesthesia in trauma medicine. Anesthesiol Res Pract.

[28] Wolmarans M, Albrecht E (2023). Regional anesthesia in the emergency department outside the operating theatre. Curr Opin Anaesthesiol.

[29] Goldsmith A, Driver L, Duggan NM (2024). Complication rates after ultrasonography-guided nerve blocks performed in the emergency department. JAMA Netw Open.

[30] Alakkad H, Naeeni A, Chan VW (2015). Infection related to ultrasound-guided single-injection peripheral nerve blockade: a decade of experience at toronto Western hospital. Reg Anesth Pain Med.

[31] Singh S, Avinash R, Jaiswal S, Kumari A (2023). Comparison of safety and efficacy of thoracic epidural block and erector spinae plane block for analgesia in patients with multiple rib fractures: a pilot single-blinded, randomised controlled trial. Indian J Anaesth.

[32] Elawamy A, Morsy MR, Ahmed MAY (2022). Comparison of thoracic erector spinae plane block with thoracic paravertebral block for pain management in patients with unilateral multiple fractured ribs. Pain Phys.

[33] O'Connell KM, Quistberg DA, Tessler R (2018). Decreased risk of delirium with use of regional analgesia in geriatric trauma patients with multiple rib fractures. Ann Surg.

[34] Liao CA, Chen YJ, Shen SJ (2024). Erector spinae plane block (ESPB) enhances hemodynamic stability decreasing analgesic requirements in surgical stabilization of rib fractures (SSRFs). World J Emerg Surg.

[35] Levochkina M, Colwell C, Maghsoudi T (2025). Should we utilize regional anesthesia for traumatic rib fracture patients? A TQIP-based study. J Surg Res.

[36] White LD, Riley B, Seidel D (2024). Rib fracture-related morbidity and mortality for older persons in the era of fascial plane blocks: a cohort study. Trauma.

[37] Easter A (2001). Management of patients with multiple rib fractures. Am J Crit Care.

[38] Bass GA, Stephen C, Forssten MP (2022). Admission triage with pain, inspiratory effort, cough score can predict critical care utilization and length of stay in isolated chest wall injury. J Surg Res.

